# Perforin and Granulysin-Mediated Cytotoxicity in Colorectal Cancer Patients

**DOI:** 10.3390/medicina62040791

**Published:** 2026-04-20

**Authors:** Ludvig Letica, Ivana Šutić Lubina, Zdrinko Brekalo, Đordano Bačić, Jelena Roganović, Ana Đorđević, Ingrid Šutić Udović, Ivona Letica, Ivana Kotri, Ines Mrakovčić-Šutić

**Affiliations:** 1 Department of Surgery, University Hospital Mostar, 88000 Mostar, Bosnia and Herzegovina; ludvigletica@gmail.com (L.L.); zdrinkobrekalo@hotmail.com (Z.B.); 2 Medical Faculty, University of Mostar, 88000 Mostar, Bosnia and Herzegovina; 3 Community Health Centre of Primorje-Gorski Kotar County, 51000 Rijeka, Croatia; ivanas222@gmail.com; 4 Department of Family Medicine, Medical Faculty, University of Rijeka, 51000 Rijeka, Croatia; 5 Department of Surgery, Clinical Hospital Centre Rijeka, 51000 Rijeka, Croatia; giordano-bacic@net.hr; 6 Department of Surgery, Medical Faculty, University of Rijeka, 51000 Rijeka, Croatia; 7 Department of Clinical Medicine 2, Faculty of Health Studies, University of Rijeka, 51000 Rijeka, Croatia; 8 Department of Pediatric Hematology and Oncology, Children’s Hospital Zagreb, 10000 Zagreb, Croatia; 9 Faculty of Biotechnology and Drug Development, University of Rijeka, 51000 Rijeka, Croatia; 10 Department of Business Development, Jadran Galenski Laboratorij, 51000 Rijeka, Croatia; ana_dordevic@hotmail.com; 11 Department of Dermatovenereology, Clinical Hospital Centre Rijeka, 51000 Rijeka, Croatia; 12 Department of Dermatovenereology, Medical Faculty, University of Rijeka, 51000 Rijeka, Croatia; 13 Department of Pediatrics, University Hospital Mostar, 88000 Mostar, Bosnia and Herzegovina; 14 University Hospital for Orthopedics and Traumatology Lovran, 51415 Lovran, Croatia; anavikotri@gmail.com; 15 Department of Physiology, Immunology and Pathophysiology, Medical Faculty, University of Rijeka, 51000 Rijeka, Croatia; 16 Department of Basic Medical Sciences, Faculty of Health Studies, University of Rijeka, 51000 Rijeka, Croatia

**Keywords:** colorectal cancer, cytotoxicity, granulysin, immunological status, perforin

## Abstract

*Background and Objectives*: The incidence of colorectal cancer (CRC) in developed Western countries is constantly growing. CRC represents the third most common cancer and the second leading cancer-related cause of death worldwide. Innate and adaptive immunity play a pivotal role in the tumor response, but many of these interactions are still not well understood. Granulysin (GNLY) is an effector, cytolytic molecule, present in human cytotoxic granules of different lymphocyte subpopulations, mainly in cytotoxic T cells and NK cells. Pore-forming proteins GNLY, perforin and granzymes play a key role in cell-mediated immune responses against tumors and infections. *Materials and Methods*: We aimed to analyze perforin and GNLY-mediated cytotoxicity in the peripheral blood of patients with CRC by flow cytometry. Simultaneously, the cells were labeled with monoclonal antibodies against perforin, GNLY and different surface antigens (CD3, CD4, CD8 and CD56). Phenotypes of lymphocyte subpopulation and expression of perforin and GNLY were analyzed using intracellular and surface immunofluorescence. *Results*: Total perforin and GNLY expressions in peripheral blood mononuclear cells (PBMC) were significantly lower than in the control group. Statistically significant differences were observed in the distribution of perforin and GNLY expression in different stages of tumors classified according to Dukes’, indicating that the percentage of total perforin and GNLY was significantly diminished in accordance with tumor progression. Perforin and GNLY expression were significantly reduced in NK and NKT cells, accompanied by reduced cytolytic potential in patients with CRC and a consequent reduction in their ability to eliminate tumors and infected cells. *Conclusions*: The determination of cytotoxic potential may provide a valuable assessment of a patient’s immune status and represent a novel therapeutic target. Patients with CRC exhibit markedly impaired perforin- and GNLY-mediated cytotoxicity that correlates with disease progression. Assessment and restoration of cytolytic potential may therefore serve as indicators of immune competence and promising therapeutic strategies to improve perioperative and oncologic outcomes.

## 1. Introduction

The incidence of colorectal cancer (CRC) in developed Western countries is constantly increasing, and research is focused on finding possible ways of earlier diagnosis, as well as potential new targets for therapy. The incidence of CRC is very high, currently ranking third in frequency of occurrence and second in mortality. In 2018, approximately 1.8 million new diagnosed cases were reported, with about 881,000 lethal outcomes [1]. Traditional, classical epidemiology investigates many risk factors such as nutrition, lifestyle, obesity, environmental and genetic factors that may lead to increasing incidence. Genetic factors include positive family history and hereditary CRC syndromes [2]. Positive family history is present in 10–20% in all cases with CRC [3]. Hereditary CRC syndromes include non-polyposis and polyposis syndromes [4]. Non-polyposis syndromes represent Lynch syndrome and familial CRC. The polyposis syndromes are more frequent and easily diagnosed, but systematic molecular analysis is necessary for proper diagnosis [5]. Environmental lifestyle risk factors are modifiable and include types of nutrition (red meat, processed meat, low intake of fruits and vegetables, deficiency of vitamins B and D) [6,7], obesity [8], reduced physical activities [9,10,11], smoking [12,13], alcohol intake [14], and others. Very important roles in the etiopathogenesis of CRC have inflammatory bowel disease (IBD) [15,16], changes in ferroptosis, necroptosis, and pyroptosis [17], diabetes mellitus type 2 [18], menopausal hormone therapy, and the use of different drugs (aspirin, statin, non-steroid anti-inflammatory drugs—NSAID). Potential protective factors should also be considered: adequate physical activity and specific nutrition (tree nuts, vitamins and calcium supplements, dietary fiber, whole grains, dairy products, fish intake) [5]. Innate and acquired immune responses represent a pivotal role in the tumor response, but many of these interactions are still not well understood. Granulysin (GNLY) is an effector, cytolytic molecule, present in human cytotoxic granules of NK and cytotoxic T cells. Together with perforin and granzymes, which also form a pore, GNLY plays a key role in cell-mediated immune responses against tumors and infections [19]. GNLY induces cell death by mitochondrial damage, accompanied by the release of cytochrome c, as well as apoptosis-inducing factors. However, these molecular mechanisms are not well defined. Cell death induced by GNLY is apoptotic, followed by the release of phosphatidylserine, which breaks the cell membrane and activates caspase 3. The apoptotic process begins with an intracellular Ca^2+^ increase and formation of mitochondrial ROS [20]. Its recombinant 9 kDa form has tumoricidal and antimicrobial activity, killing gram-positive and gram-negative bacteria, yeast, fungi and parasites, emphasizing that the killing of intracellular pathogens depends on perforin. After activation, GNLY is released, not only from NK cells, but also from both subpopulations of T cells (CD4+ and CD8+ cells). GNLY can also activate monocytes and dendritic cells [21]. In addition to the activation of GNLY in tumor and infectious diseases, its important role was also observed in the etiopathogenesis of autoimmune [22], skin [23], reproductive disorders [24], and in transplantation [25,26]. Exocytoxic release of perforin, granzymes and death receptor pathways represents a key mechanism of NK-mediated apoptotic tumor cell death [27]. Perforin initiates cell membrane invagination, granzymes delivery and, via NK cells, may control tumor development and metastasis [28,29]. Moreover, NK cells, with stimulation of death receptors, may remove target cells and induce the release of cytotoxic granules, which contain perforin and GNLY, as well as granzymes. Changes in these cytotoxic and apoptotic mechanisms represent a basic problem in all tumor types [30]. Immune escape of tumor cells may be induced by different mechanisms, which include hindering NK cell-mediated antitumor activities with cancer cells’ secretion of soluble factors or recruitment of suppressor cells [31]. Although numerous studies have been conducted on changes in immune status during colon cancer, the complexity of these etiopathogenetic mechanisms has still not yet been fully elucidated. In this work, we examined the co-expression of perforin and GNLY in patients with different stages of CRC, attempting to contribute to the elucidation of NK-mediated antitumor activity.

## 2. Materials and Methods

### 2.1. Study Design, Patients and Healthy Volunteers

A cross-sectional study involved 50 patients with newly diagnosed CRC and 40 healthy volunteers as a control group. The peripheral blood samples of CRC patients were obtained from 50 patients (ages 38–89; mean: 71 years) who underwent CRC surgical procedures at the Clinic of Abdominal Surgery, University Hospital Mostar, Bosnia and Herzegovina. Patients with CRC received 5-fluorouracil and cisplatin according to the standard protocol. They were stratified according to Dukes’ classification, which comprises four stages: Dukes’ A, B, C and D. Dukes’ A refers to tumors confined to the mucosa and/or submucosa without involvement of the muscle layer (muscularis propria). Dukes’ B includes tumors that have penetrated the muscularis propria but have not spread to regional lymph nodes. Dukes’ C denotes tumors with metastasis to regional lymph nodes. Dukes’ D represents tumors with distant metastases to other organs. The control group consisted of 40 age-matched subjects (ages 20–90; mean: 65 years).

Blood samples were collected in accordance with the International Health Guidelines in the Declaration of Helsinki “Ethical principles for medical research involving human subjects”. The study was approved by the Ethics Committee of the University Hospital Center Mostar, Bosnia and Herzegovina (No. 46/26, 21 June 2017). All subjects included in the study signed informed consent. The exclusion criteria were subjects younger than 18 years, presence of any acute or chronic inflammatory disease, immunological disease, autoimmune disorders, patients who were treated with biological, radiation or immunosuppressive therapy, or blood transfusions, as well as patients who underwent some other major surgical procedures.

### 2.2. Isolation of Peripheral Blood Mononuclear Cells

Venous peripheral blood samples of CRC patients and healthy volunteers were collected by venipuncture into “vacutainer” (Becton Dickinson, Franklin Lakes, NY, USA) and peripheral blood mononuclear cells (PBMC) were isolated on the density gradient by Lymphoprep (Nycomed Pharma AS, Oslo, Norway) and centrifugate for 20 min, at 600 G. After centrifugation, PBMC were collected and washed twice in medium (Roswell Park Memorial Institute -RPMI 1640 medium, Invitrogen, Auckland, CA, USA). PBMC were resuspended with a final concentration of 1 × 10^6^ per sample in fluorescent-activated cell sorting (FACS) buffer. Before intracellular and cell surface antigens detection, the viability of the cells was checked with propidium iodide 0.5 lg/mL per 10^6^ cells (Sigma–Aldrich, St. Louis, MO, USA) and by flow cytometric analysis (FACSCalibur, Becton Dickinson, San Jose, CA, USA) and was always >95% [32,33].

### 2.3. Simultaneous Detection of Cell Surface Antigens and Intracellular Antigen (Perforin) by Flow Cytometry

PBMC were aliquoted (10^6^ per aliquot), washed in FACS buffer and fixed in 100 μL PBS enriched with 4% paraformaldehyde, at pH 7.4 for 10 min at room temperature.

The cells were washed twice in FACS buffer and permeabilized for 20 min at room temperature with saponin buffer (0.1% saponin) (Sigma, Poole, Dorset, UK), blocking the non-specific Fc receptor binding and fixated [34,35,36]. Cells were labeled intracellularly with anti-P monoclonal antibody (Department of Physiology, Immunology and Pathophysiology, Faculty of Medicine, University of Rijeka, Rijeka, Croatia). The cells were washed twice in saponin buffer, followed by the addition of 1 mL of FACS buffer and incubation for 5 min at 22–25 °C to allow membrane restoration. Simultaneously, surface markers: CD3/CD4, CD3/CD8, CD3/CD56 were labeled with phycoerythrin-5 (Cy-PE5)-conjugated anti-CD3 (mouse UCHT1, IgG1), phycoerythrin (PE)-conjugated anti-CD4 mAb (mouse RPA-T4, IgG1), PE-conjugated anti-CD8 (mouse RPA-T8, IgG1), and PE conjugated anti-CD56 (mouse B159, IgG1) (BD Biosciences, Erembodegem, Belgium). As negative controls, isotype-matched mouse antibodies, conjugated with FITC, PE or CY-PE5 were used in the direct immunofluorescence method. All blood samples were collected and analyzed using CellQuestPro software, version 6.0. on FACSCalibur (Becton Dickinson, San Jose, CA, USA). A minimum of 10^4^ cells were analyzed.

Expression of intracellular molecule perforin was investigated in all lymphocyte subpopulations: CD3+CD56- (T lymphocytes), CD3-CD56+ (NK cells) and CD3+CD56+ (NKT cells).

### 2.4. Simultaneous Detection of Cell Surface and Intracellular Antigen (GNLY) by Flow Cytometry

The preparation of PBMC for examination of the expression of the cytotoxic molecule of GNLY was identical to that for the perforin expression investigation, except that intracellular staining was done with GNLY. In this examination, mouse IgG1 anti-human GNLY (cat. no. D-185-3; RC8 clone; MBL International, MBL Life Science, Tokyo, Japan) was added as a primary antibody and isotype-matched IgG1 (cat. no. bd554121, MOPC-21 clone; BD Pharmingen, BD Biosciences, Franklin Lakes, NJ, USA) as controls. After similar procedures like in perforin detection, surface antibody staining was performed in the same manner as double staining for perforin determination [37,38,39].

### 2.5. Statistical Analysis

The results were analyzed by program STATISTICA 12.0 (StatSoft Inc., Tulsa, OK, USA) and were presented as median (25th–75th percentile). The differences between groups were examined using the Kruskal–Wallis non-parametric test, and differences considered statistically significant at a *p* level of <0.05.

Based on the pilot study, power and sample size analyses were performed using Statistica 13 (TIBCO Software Inc., Palo Alto, CA, USA). The calculated sample size was 21 for patients with CRC and 33 for the control group, based on a two-sample t-test with a significance level of *p* < 0.05.

To investigate within-group differences, the Mann–Whitney U-test was performed. The level of significance was adjusted to account for the number of mutual comparisons. Correlation between the percentage of total perforin and total GNLY was established using Spearman’s rank correlation coefficient.

## 3. Results

### 3.1. Total Perforin and GNLY Expression in Peripheral Blood Lymphocytes

**Figure 1:**
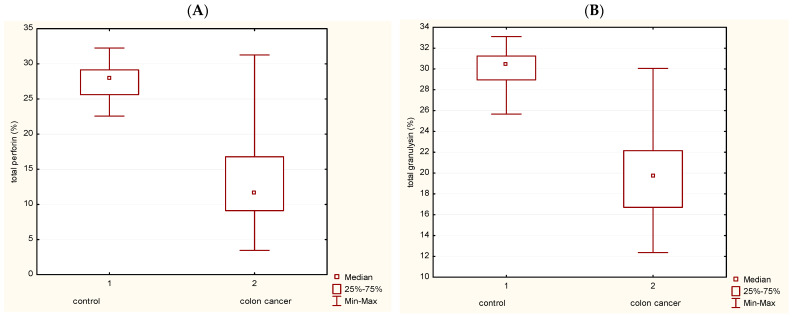
Total perforin and granulysin (GNLY) expression in peripheral blood lymphocytes (PBL). (**A**) Total perforin expression in PBL in patients with colorectal cancer and in healthy volunteers as control (*p* < 0.05). (**B**) Total GNLY expression in PBL in patients with colorectal cancer and in healthy volunteers as control (*p* < 0.05). Abbreviations: GNLY—granulysin, PBL—peripheral blood lymphocytes.

Expression of perforin (P) and GNLY within gated peripheral blood lymphocytes (PBL) was analyzed by flow cytometry (Figure 1A,B). The percentage of P+ cells in the control group was 27.46 (22.58–32.26), while in CRC patients, P+ cells were 13.33 (3.45–25.95). The expression of total perforin in CRC patients was significantly lower than in controls (Figure 1A). A non-parametric test for independent samples (Mann–Whitney U Test) was used. There is a statistically significant difference in the value of total perforin between patients with CRC and the control group (*p* < 0.05).

A statistically significant difference is also present in the expression of total GNLY (Figure 1B) between patients with CRC, where the total GNLY+ cells were 19.5 (12.36–25.85), and controls have 30.21% GNLY+ cells (25.67–32.2) and is lower in patients with CRC in comparison with controls, with statistically significant differences *p* < 0.05.

### 3.2. P Expression in Different Lymphocyte Subpopulations in Peripheral Blood Lymphocytes

**Figure 2:**
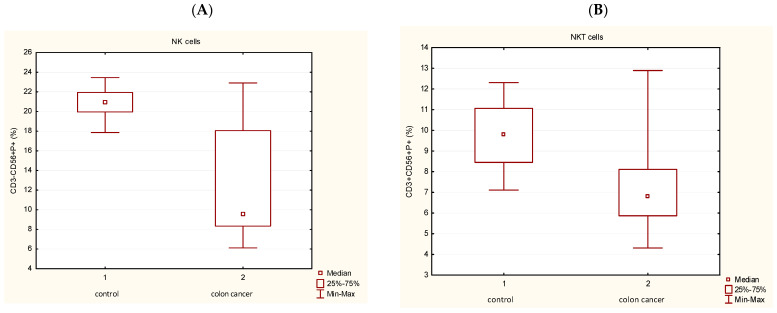
Proportion of P+ cells in the peripheral blood subpopulations. (**A**) Frequency of P+ cells in NK cells (CD3-CD56+) (*p* < 0.05). (**B**) Frequency of P+ cells in NKT cells (CD3+CD56+) (*p* < 0.05). The non-parametric test for independent samples (Mann–Whitney U Test) was used, and a statistically significant difference in the value of P+ cells was found between patients with colorectal cancer and the control group (*p* < 0.05). Abbreviations: P—perforin.

The percentage of P+ cells was examined in different lymphocyte subpopulations: in T lymphocytes (CD3+CD56-), then in T lymphocyte subsets: T helpers (CD3+CD4+), T cytotoxic cells (CD3+CD8+), NK cells CD3-CD56+) and in NKT cells (CD3+CD56+) by flow cytometry. The percentage of P+ cells in peripheral blood T lymphocytes, T helpers and T cytotoxic cells did not differ significantly among the investigated groups. However, the percentage of peripheral blood P+ cells in NK cells: CD3-CD56+P+ (Figure 2A) significantly decreased in CRC patients in comparison with controls. Similar results were in NKT cells (CD3+CD56+P+) with significantly lower percentage in CRC patients compared with controls (Figure 2B). Again, the non-parametric test for independent samples (Mann–Whitney U Test) was used, and a statistically significant difference in the value of P+ cells was found between patients with CRC and the control group (*p* < 0.05).

**Figure 3:**
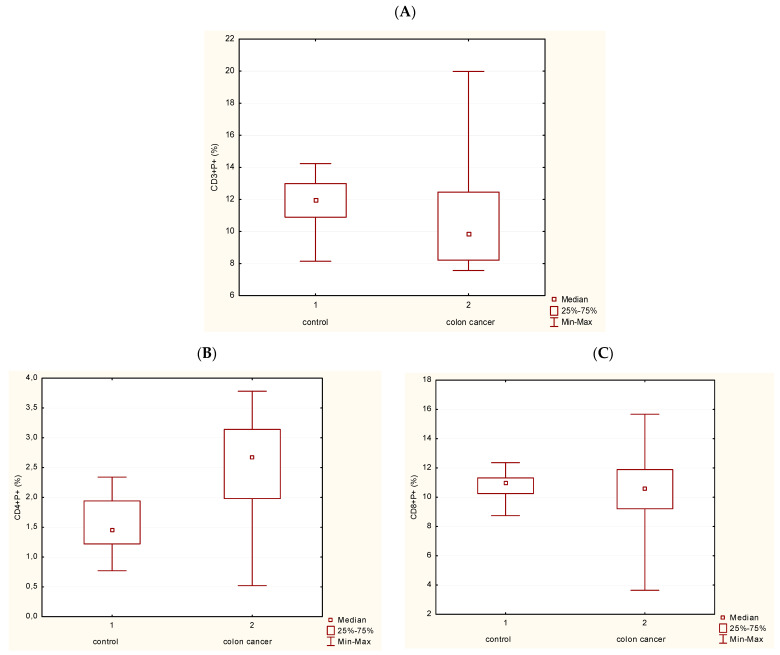
Proportion of P+ cells in the subpopulations of T cells. (**A**) Frequency of P+ cells in T cells (CD3+) (*p* < 0.05). (**B**) Frequency of P+ cells in Th cells (CD4+) (*p* < 0.05). (**C**) Frequency of P+ cells in Tc cells (CD8+). Abbreviations: P—perforin.

Figure 3 shows statistically significant differences in the frequency of P expression in T lymphocytes (CD3+CD56-) between controls and patients with CRC (Figure 3A), as well as in T helper cells (Th) (CD3+CD4+) (Figure 3B) (*p* < 0.05). Figure 3C illustrates no statistically significant differences in the frequency of P in T cytotoxic cells (Tc; CD3+CD8+) in PBL of patients with CRC compared with controls.

### 3.3. GNLY Expression in Different Lymphocyte Subpopulations in Peripheral Blood Lymphocytes

**Figure 4:**
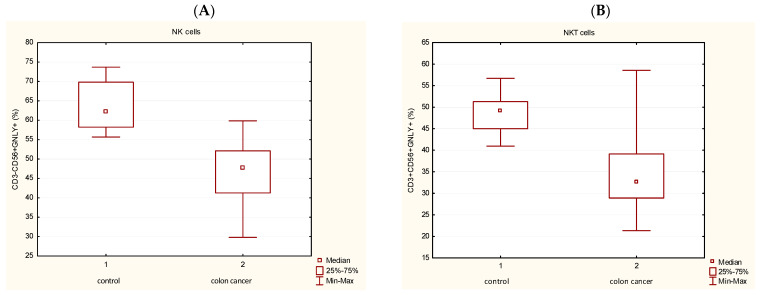
Proportion of GNLY+ cells in the peripheral blood subpopulations. (**A**) Frequency of GNLY+ cells in NK cells (CD3-CD56+) (*p* < 0.05). (**B**) Frequency of GNLY+ cells in NKT cells (CD3+CD56+) (*p* < 0.05). Mann–Whitney U Test, as a non-parametric test for independent samples, was used, and a statistically significant difference in the value of P+ cells was found between patients with colorectal cancer and the control group (*p* < 0.05). Abbreviations: GNLY—granulysin; P—perforin.

Figure 4 illustrates a diminished percentage of GNLY cells in the NK subpopulation (CD3-CD56+GNLY+) (Figure 4A) in CRC patients in comparison with controls (*p* < 0.05) and the significant decrease in NKT cells (CD3+CD56+GNLY+) in patients with CRC (Figure 4B).

**Figure 5:**
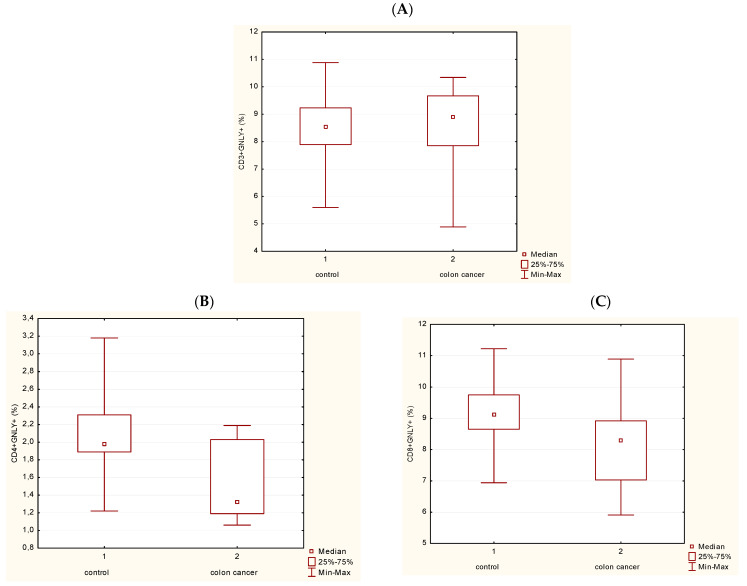
Proportion of GNLY+ cells in the subpopulations of T cells. (**A**) Frequency of GNLY+ cells in T cells (CD3+). (**B**) Frequency of GNLY+ cells in Th cells (CD4+) (*p* < 0.05). (**C**) Frequency of GNLY+ cells in Tc cells (CD8+) (*p* < 0.05). Abbreviations: GNLY—granulysin.

**Figure 6:**
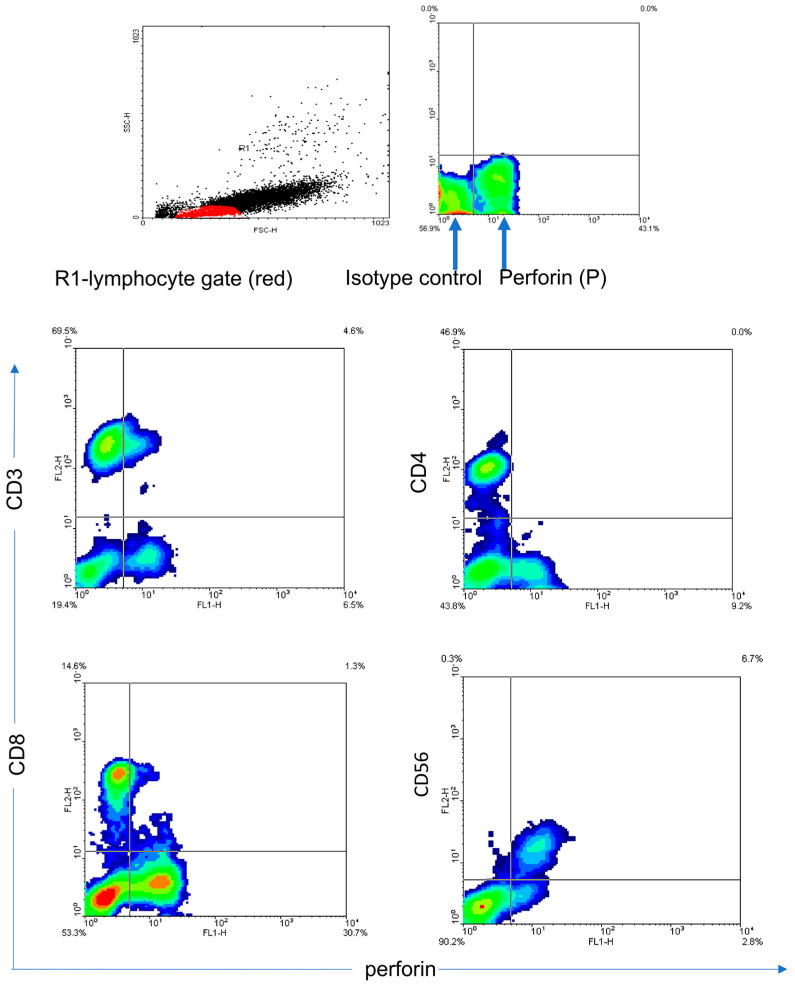
Perforin expression in the peripheral blood lymphocytes of colon cancer patients. Representative examples of contour plots showing the results from flow cytometry analyses of the R1 lymphocyte gate for P+ cells in comparison to that of the isotype control. R1 represents a lymphocyte gate (in red color). Arrows indicate the expression of perforin in comparison with isotype control. Abbreviations: P—perforin.

**Figure 7:**
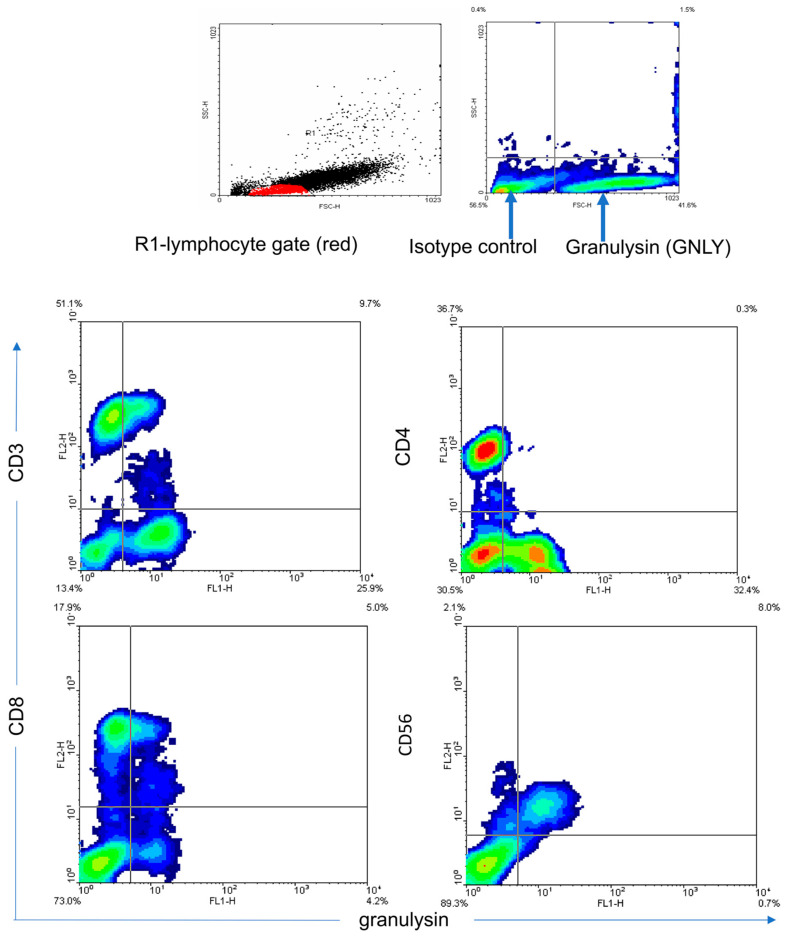
Granulysin expression in the peripheral blood lymphocytes of colon cancer patients. Representative examples of contour plots showing the results from flow cytometry analyses of the R1 lymphocyte gate for GNLY+ cells in comparison to that of the isotype control. R1 represents a lymphocyte gate (in red color). Arrows indicate the expression of GNLY in comparison with isotype control. Abbreviations: P—perforin. Abbreviations: GNLY—granulysin.

GNLY expression was analyzed in different subpopulations of T lymphocytes. Figure 5A illustrates that T lymphocytes (CD3+CD56-) do not express statistically significant changes in GNLY expression in CRC patients in comparison with controls. However, GNLY expression in Th and Tc cells is statistically diminished in CRC patients compared with controls (Figure 5B,C). A non-parametric test for independent samples (Mann–Whitney U Test) was used, and a statistically significant difference between the groups was *p* < 0.05. Figure 6 and Figure 7 show the representative contour plots in P expression (Figure 6) and in GNLY expression (Figure 7) in PBMC.

### 3.4. Distribution of P Expression in the Dukes’ Classification

**Figure 8:**
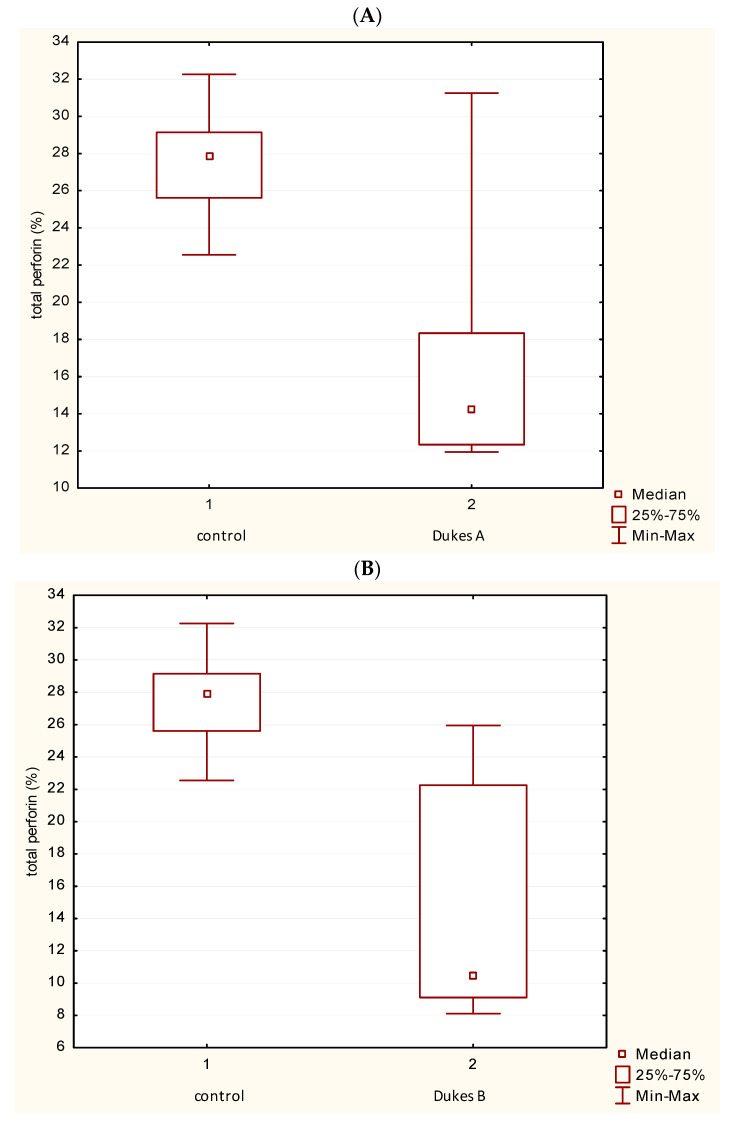

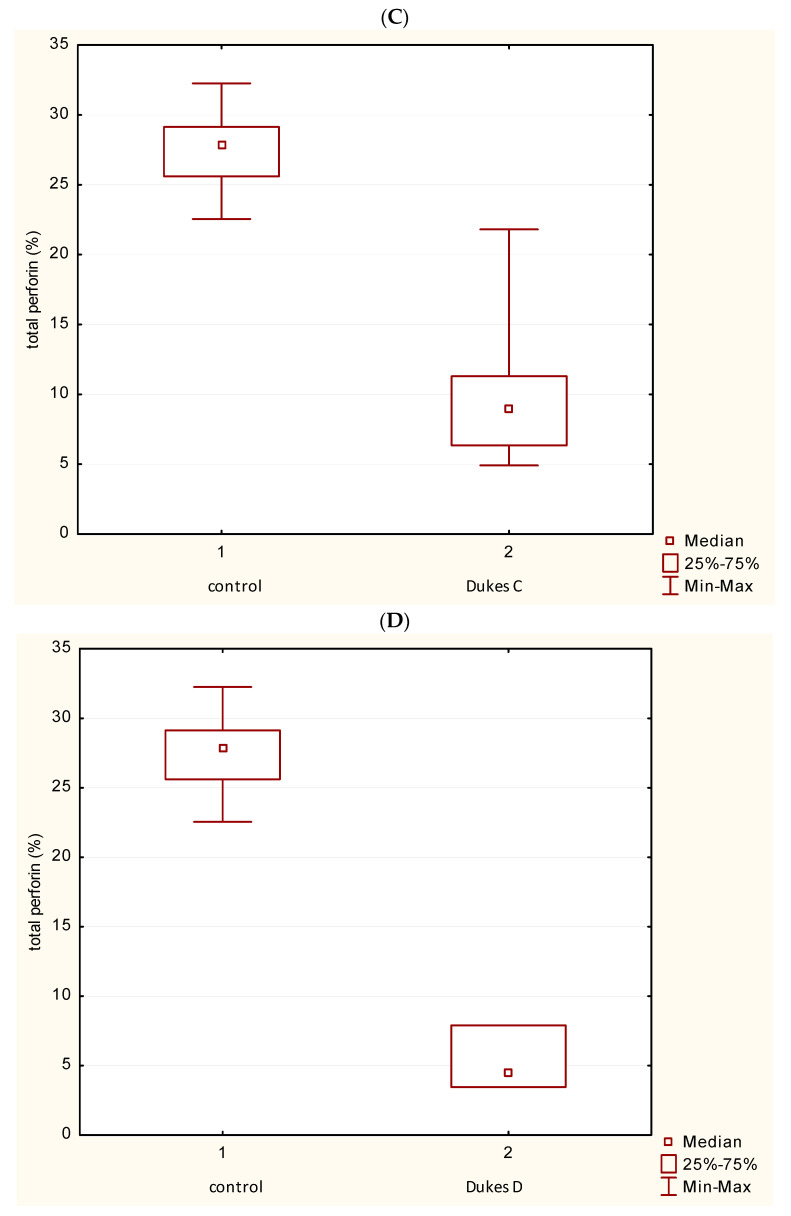

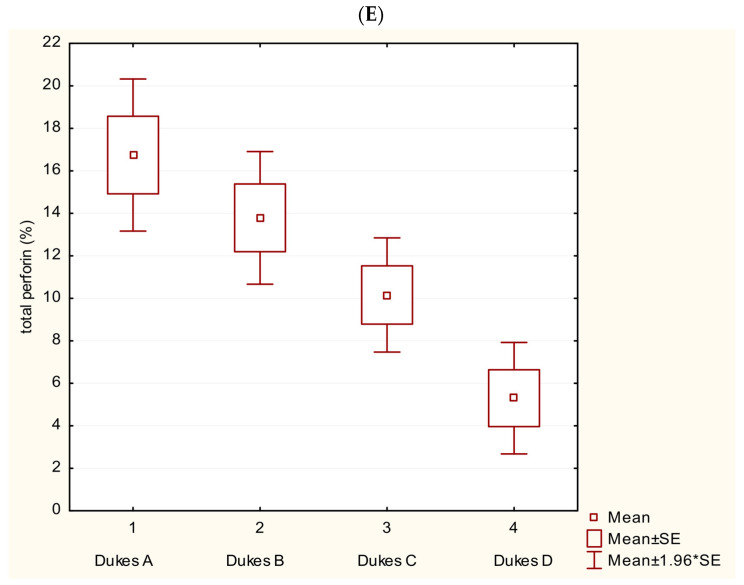
Distribution of perforin expression in the Dukes’ classification. (**A**–**D**) Total perforin (P) expression in PBL of patients with Dukes’ A, B, C and D compared with control (Mann–Whitney U test). (**E**) Statistically significant difference in the values of total perforin between Dukes’ A and Dukes’ C, Dukes’ A and Dukes’ D, Dukes’ B and Dukes’ D, with *p* < 0.05. There is no statistically significant difference between Dukes’ A and B, between Dukes’ B and C, and between Dukes’ C and D (ANOVA test).

Figure 8A–D shows a total P expression in PBL in patients with Dukes’ A, B, C and D compared with controls. Mann–Whitney U Test showed a statistically significant difference in the value of P+ cells between patients with CRC Dukes’ A, B, C and D in comparison with the control group (*p* < 0.05). Figure 8E illustrates the summary of the significance of changes in perforin expression in different stages of CRC classified by Duke. ANOVA (one-way) test was used, and it showed a statistically significant difference in the values of total perforin between Dukes’ A and Dukes’ C, Dukes’ A and Dukes’ D, Dukes’ B and Dukes’ D, with *p* < 0.05. There is no statistically significant difference between Dukes’ A and Dukes’ B, between Dukes’ B and Dukes’ C and between Dukes’ C and Dukes’ D.

### 3.5. Distribution of GNLY Expression in the Dukes’ Classification

**Figure 9:**
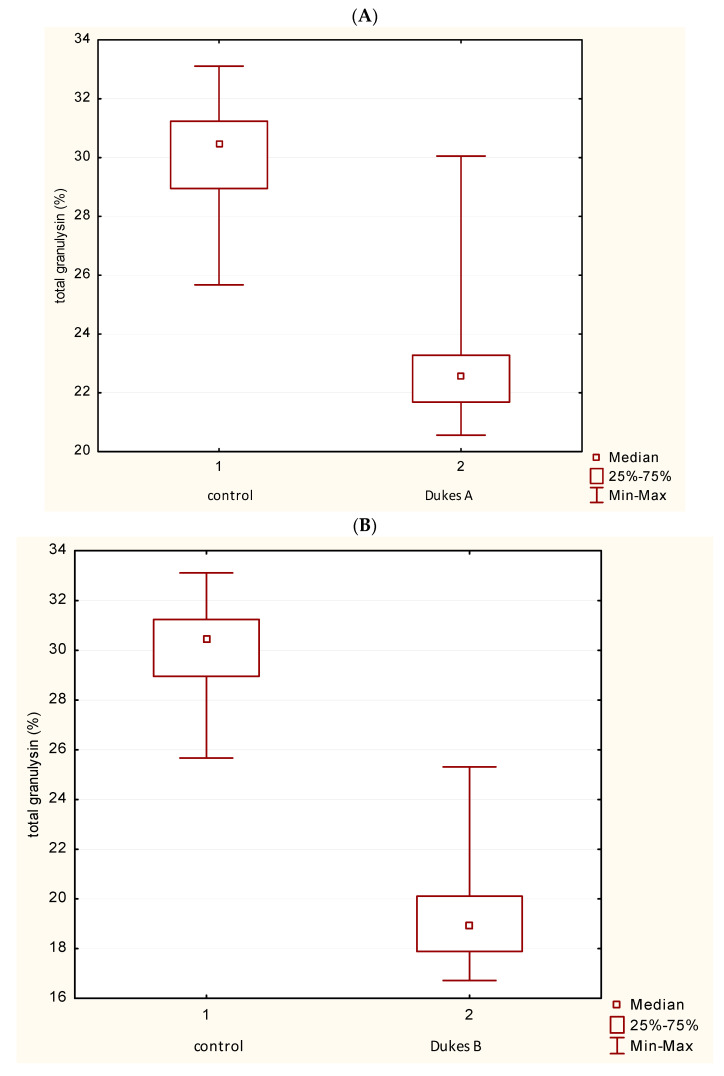

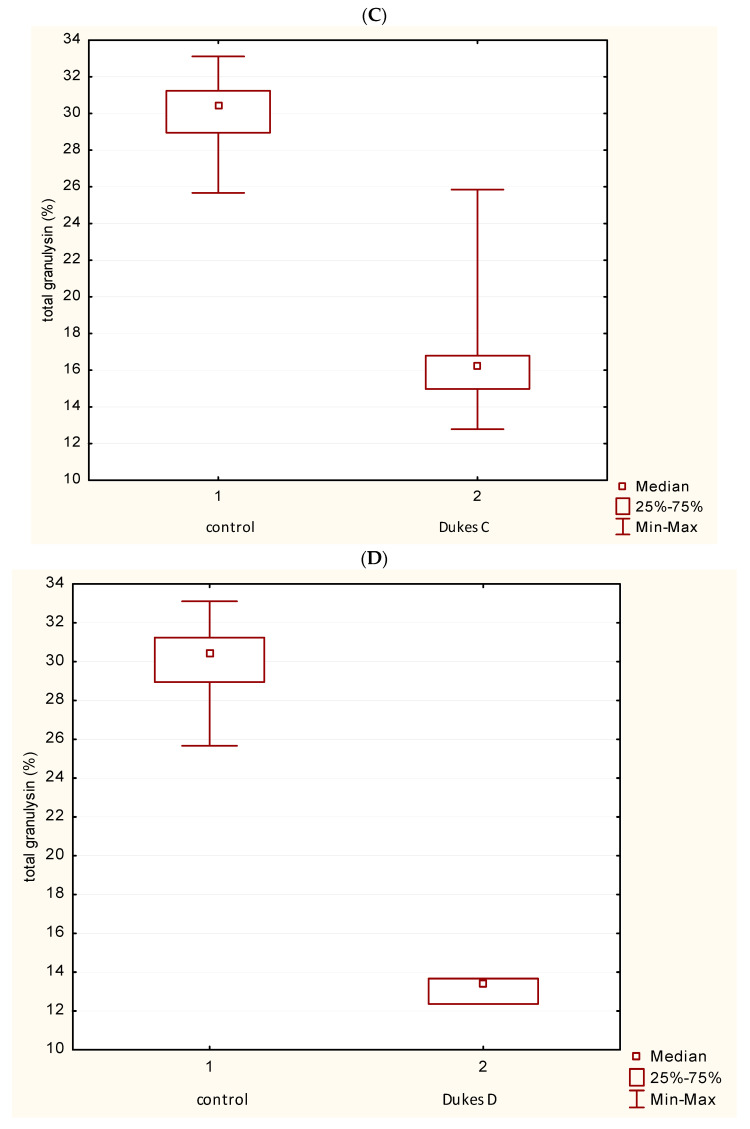

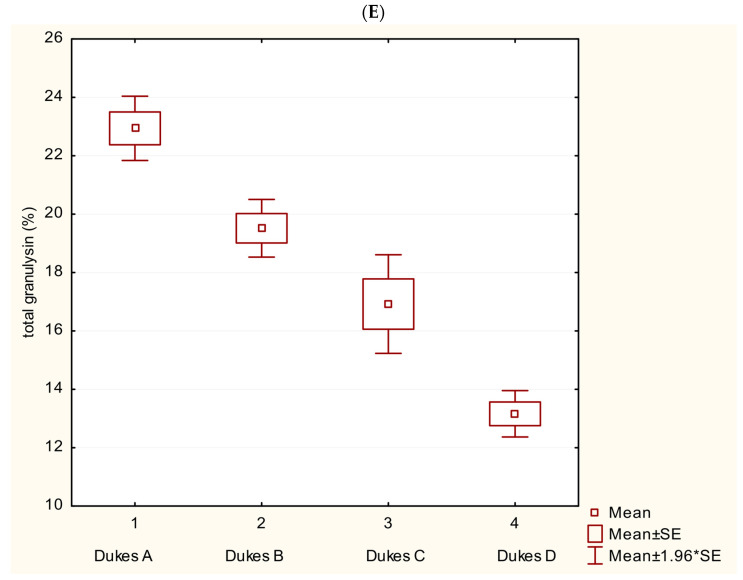
Distribution of GNLY expression in the Dukes’ classification. (**A**–**D**) Total GNLY expression in PBL in patients with Dukes’ A, B, C and D compared with controls (Mann–Whitney U test). (**E**) There are significant differences in the values of total GNLY between all 4 groups of Dukes’ (ANOVA test). Abbreviations: GNLY—granulysin.

Figure 9 illustrates the changes in GNLY expression in different stages of tumors according to Dukes’ classification. Total GNLY expression in PBL was statistically diminished in patients with Dukes’ A, B, C and D in comparison with controls. Mann–Whitney U test showed a statistically significant difference in the value of GNLY+ cells between patients with CRC Dukes’ A, B, C and D in comparison with the control group (*p* < 0.05) (Figure 9A–D). ANOVA test (one-way) was used and showed statistically significant differences in the values of total granulysin between all four Dukes’ groups (Figure 9E).

### 3.6. Correlation Between Perforin and GNLY Expression in Colorectal Cancer Patients

**Figure 10:**
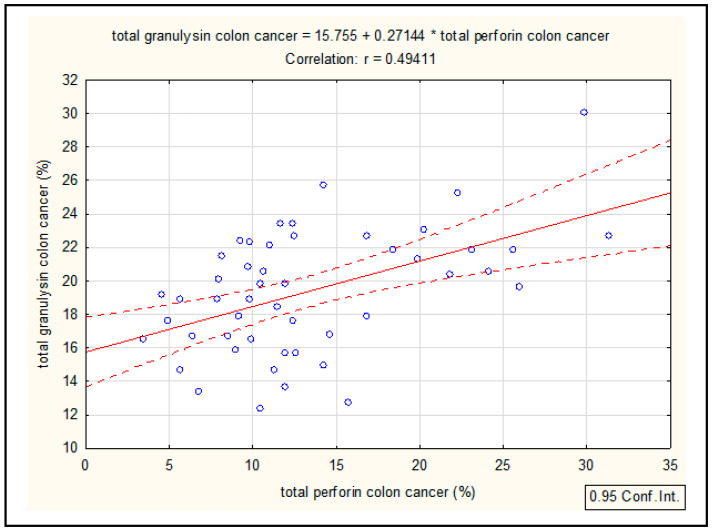
Correlation between the percentage of total perforin and total GNLY in colorectal cancer patients. Correlation between total GNLY and total perforin in patients with colorectal cancer is statistically significant (*p* < 0.05) and is r (X, Y) = 0.49, a positive and linear increase determined by Spearman’s rank correlation coefficient—abbreviation: GNLY—granulysin.

The correlation coefficient, i.e., the degree of association between the variables total GLNY and total perforin in patients with CRC, is statistically significant (*p* < 0.05) and is r (X, Y) = 0.49, indicating a good association. This is a positive and incomplete correlation, i.e., a linear increase in one variable corresponds to a linear increase in the other variable (Figure 10). Spearman’s rank correlation coefficient is used.

## 4. Discussion

This investigation includes the examination of the effects of released cytotoxic molecules, perforin and GNLY from the PBL of CRC patients. Changes in their functions may contribute to the immunologic escape in antitumor activities. Perforin is a cytotoxic molecule released from activated lymphocytes and plays a very important role in the elimination of tumor cells and virus-infected cells, usually during the effector phase of the immune response. Our obtained results show reduced values of total perforin, as well as double positive cells in peripheral blood (perforin expression is significantly reduced within NK and NKT cells. These results point to a reduced cytolytic potential of patients with colorectal tumors, and a consequent reduction in the ability to eliminate tumor cells as well as infected cells, which can lead to more frequent infections and general weakness of the organism, which is a characteristic of patients with malignancies. The low potential of cytotoxic activity was particularly observed in the low number of CD3-CD56+P+ cells. NK cells are the ones that have the highest value of perforin in healthy individuals. In patients with CRC, perforin was released from the granules, and the cells have a weakened cytolytic ability. In our previous works, we proved elevated values of regulatory T cells (Tregs) in the carcinomatous area, and we can conclude that it is a matter of dynamic changes and migration of these cells [40,41]. Tregs use important perforin/granzyme pathways to control the immune response. Contradictory data have shown that perforin and Granzyme B expressed by Tregs can inhibit tumor clearance, which contradicts the roles of these cytotoxic molecules expressed by cytotoxic T-cells and NK-cells and participating in antitumor immune responses [42]. Killer cells can fuse with the appropriate target cell, discharge cytolytic granules, including perforin, into the intercellular space. This process is dependent on the presence of Ca^2+^ ions [43]. Perforin monomers bind and polymerize in the target cell membrane, primarily around the central opening or pore. Thus, the membrane permeability of the specific target cell is disrupted, homeostasis within the cell is disrupted, endocytosis is stimulated, and osmotic lysis of these target cells is induced. Furthermore, the target cell’s damage created by the perforin pores (perforin is necessary for cytotoxicity mediated by granules) enables the communication of the target cell with mediators of cytotoxic lymphocytes, and thus perforin affects the lysis mediated by cytotoxic lymphocytes. A statistically significant difference in the distribution of perforin expression was observed across different tumor stages classified according to Dukes’. Our results indicated that the percentage of total perforin significantly diminished in accordance with tumor progression. Nakanishi H et al. [44] were the first to describe the observation that the decrease in P+ cells in CD8+ subpopulations in tumor tissues depends on tumor progression and represents the suppression of local immunity in hosts. This immune suppression represents a key problem in modern immunotherapy, and many investigators try to find possible mechanisms that may act and change this negative immune scene. Immune-activated phenotype in CRC has shown augmentation in CD8 cytotoxic T cells, as well as increased expression of perforin and granzyme B. Moreover, CRC patients with these features may have a diminished possibility of developing distant metastases [45,46]. Nowadays, dietary supplementation is very popular, and the effects of high doses of vitamin E substitution therapy in CRC patients were associated with increased NK activity. But this augmentation was not a result of increased perforin secretion nor the number of NK cells. The reason for this effect was diminished oxidative stress due to the minor increase in the induction of NKG2D expression. Vitamin E, as an antioxidant, may stimulate the function of NK cells in CRC patients. Moreover, perforin may regulate the mucosal inflammatory reaction in colitis-associated cancer [47], and our results with significantly diminished percentage of P in NK and NKT subpopulations may point this perforin function in incidence of CRC, as well as a positive correlation between the percentage of total perforin and total GNLY in which, for the first time, has shown GNLY-perforin cytotoxicity in CRC. New data try to establish GNLY as a cytotoxic agent that has the possibility to retard the in vivo development of tumors. It has been shown that GNLY-treated tumor tissues developed the augmentation of NK cell infiltration and apoptosis [48]. It is well known that GNLY is a cytotoxic molecule present in NK cells, as well as in cytotoxic T cells, and is responsible for cell-mediated immune response against tumor growth and infection. Our data have shown that total GNLY expression is significantly lower in patients with CRC, indicating that the cytotoxic ability of PBL is significantly reduced in these patients, and may be associated with a worse outcome and a weaker response to therapy. Cytolytic molecules may be involved in endoplasmic reticulum membrane permeability and may act on the augmentation of the intracellular Ca^2+^ levels, together with the production of ROS and stimulation of the changes in mitochondrial membrane potential [49]. This intracellular cascade of activation contributes to the development of the caspase-dependent apoptosis, via cytochrome c release and some other pro-apoptotic factors. GNLY expression is reduced in all tumor types classified by Dukes’, and the reduction is proportional to the stages of the disease. Weakened immune response represents one of the biggest challenges in the therapy of patients with CRC, and any knowledge that helps in understanding complex immune events can contribute to faster diagnosis and more effective treatment.

The expression of the *GNLY* gene may represent a good diagnostic biomarker, together with some other genes such as *SLC6A6* (Solute-Carrier Family 6 Member 6), *LCK* (LCK Proto-Oncogene, Src Family Tyrosine Kinase) and *LAMP2* (Lysosomal Associated Membrane Protein 2) in the first stage of CRC [50,51]. Anticancer immunity may include increased activity of cytotoxic T cells, followed by elevated release of perforin and granzymes, augmentation of macrophages and Tregs in the tumor microenvironment [52]. Our findings with a positive and statistically significant correlation between total GNLY and total perforin in patients with CRC may indicate a useful tool in the diagnosis and/or prognosis of the disease. The correlation between perforin and GNLY cytolytic activities and their interactions with the tumor stages still has not been examined, and the possible effects on the inflammatory response pathway and anticancer immunity are still not well understood. Further investigations are needed to elucidate these interactions.

## 5. Conclusions

Patients with malignancies develop immune suppression, which represents a major problem for effective immunotherapy. GNLY and perforin cytotoxic potential is severely impaired in CRC patients and indicates the importance of restoring cytolytic potential as one of the possible therapeutic targets. Preserved cytolytic potential may diminish the intraoperative risk, contribute to better postoperative recovery, immediately after the operation and later during postoperative oncology therapy. Diminished expression of perforin and GNLY in patients with CRC, along with reduced expression of these cytotoxic molecules in NK and NKT cells, correlates with cancer progression, and the determination of cytotoxic potential may prove to be a good assessment of the patient’s immune status, and they may represent a new targeted therapy.

## Data Availability

The data presented in this study are available on request from the corresponding authors. The data is not publicly available due to privacy and ethical restrictions.

## Abbreviations

The following abbreviations are used in this manuscript:

CRC	Colorectal cancer
FACS	Fluorescent-activated cell sorting
GNLY	Granulysin
IBD	Inflammatory bowel disease
NSAID	Non-steroid anti-inflammatory drugs
PBL	Peripheral blood lymphocytes
PBMC	Peripheral blood mononuclear cells

## References

[1] Baidoun F., Elshiwy K., Elkeraie Y., Merjaneh Z., Khoudari G., Sarmini M.T., Gad M., Al-Husseini M., Saad A. (2021). Colorectal Cancer Epidemiology: Recent Trends and Impact on Outcomes. Curr. Drug Targets.

[2] Anderson A.S., Caswell S., Macleod M., Steele R.J.C., Berg J., Dunlop J., Stead M., Eadie D., O’Carroll R.E. (2017). Health behaviors and their relationship with disease control in people attending genetic clinics with a family history of breast or colorectal cancer. J. Genet. Couns..

[3] Parisi A., Cortellini A., Venditti O., Santo V., Sidoni T., Cannita K., Ficorella C., Porzio G. (2020). Family history of cancer as potential prognostic factor in stage II colorectal cancer: A retrospective monoinstitutional study. J. Gastrointest. Cancer.

[4] Sninsky J.A., Shore B.M., Lupu G.V., Crockett S.D. (2022). Risk Factors for Colorectal Polyps and Cancer. Gastrointest. Endosc. Clin. N. Am..

[5] Dekker E., Tanis P.J., Vleugels J.L.A., Kasi P.M., Wallace M.B. (2019). Colorectal cancer. Lancet.

[6] Farvid M.S., Sidahmed E., Spence N.D., Mante Angua K., Rosner B.A., Barnett J.B. (2021). Consumption of red meat and processed meat and cancer incidence: A systematic review and meta-analysis of prospective studies. Eur. J. Epidemiol..

[7] Thanikachalam K., Khan G. (2019). Colorectal Cancer and Nutrition. Nutrients.

[8] Kyrgiou M., Kalliala I., Markozannes G., Cividini S., Gunter M.J., Nautiyal J., Gabra H., Paraskevaidis E., Martin-Hirsch P., Tsilidis K.K. (2017). Adiposity and cancer at major anatomical sites: Umbrella review of the literature. BMJ.

[9] Song M., Chan A.T., Sun J. (2020). Influence of the gut microbiome, diet, and environment on risk of colorectal cancer. Gastroenterology.

[10] Kerr J., Anderson C., Lippman S.M. (2017). Physical activity, sedentary behaviour, diet, and cancer: An update and emerging new evidence. Lancet Oncol..

[11] Ho M., Ho J.W.C., Fong D.Y.T., Lee C.F., Macfarlane D.J., Cerin E., Lee A.M., Leung S., Chan W.Y.Y., Leung I.P.F. (2020). Effects of dietary and physical activity interventions on generic and cancer-specific health-related quality of life, anxiety, and depression in colorectal cancer survivors: A randomized controlled trial. J. Cancer Surviv..

[12] Bai X., Wei H., Liu W., Coker O.O., Gou H., Liu C., Zhao L., Li C., Zhou Y., Wang G. (2022). Cigarette smoke promotes colorectal cancer through modulation of gut microbiota and related metabolites. Gut.

[13] Marafini I., Monteleone G. (2023). Smoking and colorectal cancer in inflammatory bowel disease: Quantity matters?. United Eur. Gastroenterol. J..

[14] Ionescu V.A., Gheorghe G., Bacalbasa N., Chiotoroiu A.L., Diaconu C. (2023). Colorectal Cancer: From Risk Factors to Oncogenesis. Medicina.

[15] Shah S.C., Itzkowitz S.H. (2022). Colorectal Cancer in Inflammatory Bowel Disease: Mechanisms and Management. Gastroenterology.

[16] Xia Y., Zhang L., Ocansey D.K.W., Tu Q., Mao F., Sheng X. (2023). Role of glycolysis in inflammatory bowel disease and its associated colorectal cancer. Front. Endocrinol..

[17] Zhou P., Zhang S., Wang M., Zhou J. (2023). The Induction Mechanism of Ferroptosis, Necroptosis, and Pyroptosis in Inflammatory Bowel Disease, Colorectal Cancer, and Intestinal Injury. Biomolecules.

[18] Lawler T., Walts Z.L., Steinwandel M., Lipworth L., Murff H.J., Zheng W., Warren Andersen S. (2023). Type 2 Diabetes and Colorectal Cancer Risk. JAMA Netw. Open.

[19] Al-Wasaby S., Guerrero-Ochoa P., Ibáñez-Pérez R., Soler R., Conde B., Martínez-Lostao L., Anel A. (2021). In vivo potential of recombinant granulysin against human melanoma. Cancer Treat. Res. Commun..

[20] Iranpour S., Arif M., Szegezdi E. (2025). Disrupting membranes, controlling cell fate: The role of pore-forming proteins in cell death and therapy. Apoptosis.

[21] Liu X., Lieberman J. (2020). Knocking ’em dead: Pore-forming proteins in immune defense. Annu. Rev. Immunol..

[22] Genç G.C., Çelik S.K., Arpaci D., Aktaş T., Can M., Bayraktaroğlu T., Dursun A. (2022). Granulysin peptide and gene polymorphism in the pathogenesis of Hashimoto thyroiditis. Acta Endocrinol..

[23] Wang C.W., Wu M.Y., Chen C.B., Lin W.C., Wu J., Lu C.W., Chen W.T., Wang F.Y., Hui R.C., Chi M.H. (2023). Clinical characteristics and immune profiles of patients with immune-mediated alopecia associated with COVID-19 vaccinations. Clin. Immunol..

[24] Gulić T., Laskarin G., Dominović M., Glavan Gacanin L., Babarović E., Rubeša Ž., Haller H., Rukavina D. (2018). Granulysin-mediated apoptosis of trophoblasts in blighted ovum and missed abortion. Am. J. Reprod. Immunol..

[25] Jung J.M., Won C.H., Chang S.E., Lee M.W., Lee W.J. (2025). Spatially resolved single-cell transcriptome analysis of mycosis fungoides reveals distinct biomarkers GNLY and FYB1 compared with psoriasis and chronic spongiotic dermatitis. Mod. Pathol..

[26] Soleimanian S., Yaghobi R., Karimi M.H., Geramizadeh B., Roozbeh J. (2023). Altered signatures of plasma inflammatory proteins and phenotypic markers of NK cells in kidney transplant patients upon CMV reactivation. Curr. Microbiol..

[27] Sordo-Bahamonde C., Lorenzo-Herrero S., Payer Á.R., Gonzalez S., López-Soto A. (2020). Mechanisms of Apoptosis Resistance to NK Cell-Mediated Cytotoxicity in Cancer. Int. J. Mol. Sci..

[28] O’Neill K., Pastar I., Tomic-Canic M., Strbo N. (2020). Perforins expression by cutaneous gamma delta T cells. Front. Immunol..

[29] Krawczyk P.A., Laub M., Kozik P. (2020). To kill but not be killed: Controlling the activity of mammalian pore-forming proteins. Front. Immunol..

[30] Guan X., Guo H., Guo Y., Han Q., Li Z., Zhang C. (2024). Perforin 1 in cancer: Mechanisms, therapy, and outlook. Biomolecules.

[31] Sordo-Bahamonde C., Vitale M., Lorenzo-Herrero S., López-Soto A., Gonzalez S. (2020). Mechanisms of resistance to NK cell immunotherapy. Cancers.

[32] Banias L., Jung I., Chiciudean R., Gurzu S. (2022). From Dukes’-MAC staging system to molecular classification: Evolving concepts in colorectal cancer. Int. J. Mol. Sci..

[33] Mrakovcic-Sutic I., Tokmadzic V.S., Laskarin G., Mahmutefendic H., Lucin P., Zupan Z., Sustic A. (2010). Early changes in frequency of peripheral blood lymphocyte subpopulations in severe traumatic brain-injured patients. Scand. J. Immunol..

[34] Sotosek S., Sotosek Tokmadzic V., Mrakovcic-Sutic I., Tomas M.I., Dominovic M., Tulic V., Sutic I., Maricic A., Sokolic J., Sustic A. (2011). Comparative study of frequency of different lymphocyte subpopulations in peripheral blood of patients with prostate cancer and benign prostatic hyperplasia. Wien. Klin. Wochenschr..

[35] Grbas H., Mrakovcić-Sutić I., Depolo A., Radosević-Stasić B. (2009). Perforin expression in peripheral blood lymphatic cells of patients subjected to laparoscopic or open cholecystectomy. Mediat. Inflamm..

[36] Sotosek Tokmadzic V., Laskarin G., Mahmutefendic H., Lucin P., Mrakovcic-Sutic I., Zupan Z., Sustic A. (2012). Expression of cytolytic protein perforin in peripheral blood lymphocytes in severe traumatic brain injured patients. Injury.

[37] Tokmadžić V.S., Tomaš M.I., Sotošek S., Laškarin G., Dominović M., Tulić V., Dorđević G., Sustić A., Mrakovčić-Šutić I. (2011). Different perforin expression in peripheral blood and prostate tissue in patients with benign prostatic hyperplasia and prostate cancer. Scand. J. Immunol..

[38] Drvar V., Ćurko-Cofek B., Karleuša L., Aralica M., Rogoznica M., Kehler T., Legović D., Rukavina D., Laskarin G. (2022). Granulysin expression and granulysin-mediated apoptosis in the peripheral blood of osteoarthritis patients. Biomed. Rep..

[39] Dominovic M., Laskarin G., Glavan Gacanin L., Haller H., Rukavina D. (2016). Colocalization of granulysin protein forms with perforin and LAMP-1 in decidual lymphocytes during early pregnancy. Am. J. Reprod. Immunol..

[40] Crespo Â.C., Mulik S., Dotiwala F., Ansara J.A., Sen Santara S., Ingersoll K., Ovies C., Junqueira C., Tilburgs T., Strominger J.L. (2020). Decidual NK cells transfer granulysin to selectively kill bacteria in trophoblasts. Cell.

[41] Zheng Z., Wieder T., Mauerer B., Schäfer L., Kesselring R., Braumüller H. (2023). T cells in colorectal cancer: Unravelling the function of different T cell subsets in the tumor microenvironment. Int. J. Mol. Sci..

[42] Wang Y., Sedimbi S., Löfbom L., Singh A.K., Porcelli S.A., Cardell S.L. (2018). Unique invariant natural killer T cells promote intestinal polyps by suppressing TH1 immunity and promoting regulatory T cells. Mucosal Immunol..

[43] Tibbs E., Kandy R.R.K., Jiao D., Wu L., Cao X. (2023). Murine regulatory T cells utilize granzyme B to promote tumor metastasis. Cancer Immunol. Immunother..

[44] Naneh O., Kozorog M., Merzel F., Gilbert R., Anderluh G. (2023). Surface plasmon resonance and microscale thermophoresis approaches for determining the affinity of perforin for calcium ions. Front. Immunol..

[45] Koelzer V.H., Sokol L., Zahnd S., Christe L., Dawson H., Berger M.D., Inderbitzin D., Zlobec I., Lugli A. (2017). Digital analysis and epigenetic regulation of the signature of rejection in colorectal cancer. Oncoimmunology.

[46] Dong Y., Liu Y., Shu Y., Chen X., Hu J., Zheng R., Ma D., Yang C., Guan X. (2017). Link between risk of colorectal cancer and serum vitamin E levels: A meta-analysis of case-control studies. Medicine.

[47] Zhang M., Li X., Zhang Q., Yang J., Liu G. (2023). Roles of macrophages on ulcerative colitis and colitis-associated colorectal cancer. Front. Immunol..

[48] Ibáñez-Pérez R., Guerrero-Ochoa P., Al-Wasaby S., Navarro R., Tapia-Galisteo A., De Miguel D., Gonzalo O., Conde B., Martínez-Lostao L., Hurtado-Guerrero R. (2019). Anti-tumoral potential of a human granulysin-based, CEA-targeted cytolytic immunotoxin. OncoImmunology.

[49] Díaz-Basabe A., Burrello C., Lattanzi G., Botti F., Carrara A., Cassinotti E., Caprioli F., Facciotti F. (2021). Human intestinal and circulating invariant natural killer T cells are cytotoxic against colorectal cancer cells via the perforin–granzyme pathway. Mol. Oncol..

[50] Janikowska G., Janikowski T., Pyka-Pająk A., Mazurek U., Janikowski M., Gonciarz M., Lorenc Z. (2018). Potential biomarkers for the early diagnosis of colorectal adenocarcinoma—Transcriptomic analysis of four clinical stages. Cancer Biomark..

[51] Narayanan S., Kawaguchi T., Yan L., Peng X., Qi Q., Takabe K. (2018). Cytolytic activity score to assess anticancer immunity in colorectal cancer. Ann. Surg. Oncol..

[52] Lo Bello G., Akarca A.U., Ambrosio M.R., Agostinelli C., Molina-Kirsch H., Ramsay A., Rodriguez-Justo M., Pugh M., Zhao S., DeLisser M. (2018). Granulysin, a novel marker for extranodal NK/T cell lymphoma, nasal type. Virchows Arch..

